# Supporting Weight Management for Kidney Transplant Candidates in People With Obesity on Haemodialysis: The First National Survey of UK Kidney Healthcare Providers

**DOI:** 10.1111/dom.70900

**Published:** 2026-05-31

**Authors:** Adrian Brown, Helen L. MacLaughlin, Kieran McCafferty, Sebastian Potthoff, Sharlene Greenwood, Victoria Vickerstaff, Rachel L. Batterham, Rachel Thomas, Claudia Hobbs, Reza Motallebzadeh

**Affiliations:** ^1^ Centre for Obesity Research University College London London UK; ^2^ Bariatric Centre for Weight Management and Metabolic Surgery University College London Hospital NHS Trust London UK; ^3^ National Institute of Health and Care Research University College London Hospitals Biomedical Research Centre London UK; ^4^ School of Exercise and Nutrition Sciences Queensland University of Technology Brisbane Australia; ^5^ Nutrition Research Collaborative Royal Brisbane and Women's Hospital Brisbane Australia; ^6^ Department of Renal Medicine Royal London Hospital London UK; ^7^ School of Communities and Education Northumbria University Newcastle Newcastle Upon Tyne UK; ^8^ Department of Renal Medicine King's College Hospital NHS Trust London UK; ^9^ Renal Sciences, Faculty of Life Sciences and Medicine King's College London London UK; ^10^ The Centre for Methodology and Evaluation Queen Mary University of London London UK; ^11^ International Medical Affairs, Eli Lilly Basingstoke UK; ^12^ Edinburgh Transplant Centre Royal Infirmary Edinburgh Edinburgh Scotland; ^13^ Department of Nephrology and Transplantation Royal Free London NHS Foundation Trust London UK; ^14^ Research Department of Surgical Biotechnology, Division of Surgery & Interventional Sciences University College London London UK

**Keywords:** barriers, incretin therapy, obesity care, perceptions, real‐world evidence, weight management

## Abstract

**Aims:**

Access to kidney transplantation (KTx) for people living with obesity and kidney failure is often challenging, especially given the inconsistent availability of publicly funded obesity services across the United Kingdom. This study explored UK kidney healthcare providers' views on weight management support for patients with obesity on haemodialysis before KTx, criteria for transplant waitlisting and the potential presence of weight stigma in clinical settings.

**Materials and Methods:**

An online national UK cross‐sectional survey was conducted (July–December 2024), using purposive and snowball sampling of kidney/transplant centres. Weight bias was assessed using the Fat Phobia Scale (F‐Scale). Descriptive and multivariable regression analyses were conducted.

**Results:**

In total, 227 respondents took part, representing 78.6% of UK kidney centres. Most providers (93%) reported that access to transplantation was the main motivator for patient weight loss, while major challenges included poor access to obesity services (61.7%), long waiting times (59.9%) and limited funding (58.6%). Only 60.1% had access to any obesity service, and referrals to bariatric/metabolic surgery (38.9%) and use of incretin‐based therapies (34.7%) were uncommon. KTx waitlisting criteria varied widely, the most common BMI cut‐off was ≤ 35 kg/m^2^. Weight stigma was prevalent, with a mean F‐Scale score of 3.42 and 99.5% showing negative attitudes towards obesity.

**Conclusions:**

This study provides the first UK‐wide overview of practice demonstrating inconsistent access to obesity treatment services, BMI‐based listing criteria and presence of weight stigma. These data underscore the urgent need for improved obesity care and equitable access to transplantation.

## 1 Introduction

Obesity affects approximately 28% of adults in the United Kingdom [1] and is associated with progression to kidney failure (KF) [2, 3], resulting in patients requiring kidney replacement therapy (KRT). Kidney transplantation (KTx) remains the preferred KRT option in KF due to lower costs compared to dialysis, improved quality of life and better long‐term survival [4]. Global data suggests 6%–30% of adults with chronic kidney disease (CKD) [5] live with obesity, while figures in KF and KTx candidates are higher (39% and 46%, respectively) [6]. However inequitable access to KTx for haemodialysis (HD) patients living with obesity (PLwO) persists, with up to 30% excluded; despite international guidelines recommending obesity should not preclude KTx [7, 8]. While obesity can increase the risk of surgical complications after KTx [9], graft survival appears not to differ across BMI categories (up to BMI ≥ 40 kg/m^2^), with patient survival generally superior following KTx to remaining on HD [10, 11, 12, 13, 14, 15].

To be eligible for KTx, PLwO are often required to lose weight to meet the listing cutoff criteria set out by certain transplantation centres [7, 8], though evidence suggests access to obesity services remains challenging [16]. Notwithstanding multiple approaches being available including diet, physical activity, incretin‐based therapies (e.g., Semaglutide, Tirzepatide) and metabolic/bariatric surgery [17, 18]; there remains limited high‐quality evidence of effective weight loss strategies in PLwO and KF, particularly patients on HD [19].

Most studies on access to obesity services and KTx listing criteria for PLwO have focused on BMI listing requirements, drawn views of only a small subset of the kidney MDT (typically nephrologists, surgeons and dietitians) and been conducted mainly in the North America [20, 21, 22, 23, 24, 25, 26], limiting their relevance to the United Kingdom. Furthermore, no study has examined weight stigma in kidney healthcare providers (HCPs). Therefore, this study aimed to explore healthcare providers' perspectives on how PLwO and KF on HD are supported to lose weight before KTx, the weight‐related criteria used for KTx listing and whether weight stigma may be present within publicly‐funded healthcare services in the United Kingdom.

## 2 Materials and Methods

The study was a cross‐sectional survey of UK kidney centres. The survey was hosted on REDCap in Data Safe Haven at University College London, an encrypted secure online platform. Inclusion criteria were that participants were UK HCPs at general nephrology and/or kidney transplant centres and aged between 18 and 70 years. All members of staff within the kidney centres were eligible to complete the survey. Exclusion criteria were HCPs working outside the United Kingdom and those not willing to give informed consent. The study was granted ethical approval by the UCL Research Ethics Committee (REC number 16191/009).

Kidney and transplant centres were identified via the UK Renal Registry. Centres were approached via national/local professional and patient kidney groups using existing professional networks and distribution lists. In addition, service contacts were identified by accessing publicly available contact details on websites and social media including National Health Service (NHS) Trust websites, Facebook and X to help identify eligible HCPs. All survey responses were anonymous as no identifiable information was collected.

Eligible participants were sent a survey link either through an email invitation or social media post. Once potential participants accessed the survey (see Supporting Information for survey and development process), they were asked to read a participant information sheet, followed by giving electronic informed consent. They then answered a series of questions lasting approximately 15 min. Demographic data including professional role, age, ethnicity, gender and country of work were collected. This was followed by a series of questions to understand what was offered to PLwO and KF on HD within their centres to lose weight, the referral listing criteria for KTx and finally a validated measure to assess explicit weight stigma, the Fat‐Phobia Scale—Short Form (F‐Scale).

The F‐Scale [27] is a 14‐item measure of explicit weight bias that presents weight‐related stereotypes as well as the opposite (e.g., fast and slow), where participants indicate their perceptions of PLwO using a five point Likert response scale. Higher scores indicate higher fat phobia. Average scores for the 14 items were calculated (1–5), with a score of 2.5 indicating a neutral attitude, a score < 2.5 indicating a positive attitude and a score > 2.5 indicating a negative attitude [28]. Further categories within the F‐Scale are mild (2.51–3.45), moderate (3.46–4.39) and high (≥ 4.4) fat phobia levels [29, 30].

### 2.1 Specialist Weight Management Services

To aid context, the services described within this study refer to publicly funded services within the NHS in the United Kingdom. Specialist weight management services are obesity treatment services led by a multidisciplinary team (MDT) offering a combination of medical, dietary, behavioural and surgical interventions [31].

### 2.2 Data Analysis

The statistical plan was formulated and analyses conducted by the study statistician (V.V.) alongside sample size calculation (see Supporting Information). Patient characteristics were summarised using means and standard deviation (SD) or medians and interquartile ranges (IQR) for continuous measures. Categorical variables were described using counts and percentages. Chi‐squared tests, Fisher's exact tests and *t*‐tests were performed to compare the outcomes of the survey between the different UK countries devolved nations (England, Northern Ireland [NI], Scotland and Wales), participant job role (medical [i.e., surgeon and nephrologist] compared to other HCPs) and whether the centre was a transplant centre or referral centre. As the survey covered multiple domains with varied question formats rather than a single construct, reliability metrics, apart from the F‐Scale, were not measured, alongside test–retest reliability as the survey was not intended for repeated measurement.

To identify predictors of weight stigma, as measured by the F‐Scale, univariable linear regression analyses were performed. Variables showing potential associations in the univariable regression analyses (*p* < 0.1) were subsequently included in a multivariable linear regression model. A stepwise selection procedure was used, using a backward elimination, with variables retained in the final model if they met the significance threshold of *p* < 0.05. Due to the limited numbers of participants from ethnic minority groups, ethnicity was dichotomised (‘White’ and ‘Ethnic Minority’). Regression coefficients, 95% confidence intervals (CIs) and *p* values were reported for outcomes. Model assumptions were checked. All statistical analyses were performed in Stata (version 18). No correction for multiple comparisons were applied to subgroup analyses across devolved nations, roles and centre types, as these were exploratory and this survey was not designed as a confirmatory study. Statistical significance was defined as *p* < 0.05.

## 3 Results

Between July and December 2024, 78.6% of UK kidney centres responded (Table 1; full listing of UK kidney centres and responses are in Tables S1–S4). A total of 238 HCPs responded to the survey. Eleven were removed either for withdrawing consent (*n* = 1) or having incomplete data (*n* = 10), with 227 participants included in the final analysis.

**TABLE 1:** Demographic characteristics of the participants (*n* = 227).

Characteristics, *n* (%)	*n* = 227
Gender, *n* (%)	
Male	49 (21.6)
Women	176 (77.5)
Prefer not to say	2 (0.9)
Age (years), mean (SD)	44.6 (10.5)
Ethnicity, *n* (%)	
Asian	27 (11.9)
Black	2 (0.9)
Mixed	4 (1.8)
Other	5 (1.3)
White	186 (81.9)
Prefer not to say	5 (2.2)
Years since registration, median (IQR)	15.0 (6.0, 20.4)
Role within the service, *n* (%)	
Dietitian	77 (33.9)
Nephrologist	50 (22.0)
Psychologist/psychiatrist	7 (3.1)
Nurse	49 (21.6)
Surgeon	14 (6.2)
Physiotherapist	8 (3.5)
OT	3 (1.3)
Pharmacist	6 (2.6)
Counsellor	2 (0.9)
Other	13 (5.7)
What patients do you support in yourprofessional role, *n* (%)	
Haemodialysis	208 (91.6)
Peritoneal dialysis	138 (60.8)
Transplant	144 (63.4)
Advanced kidney care	150 (66.1)
General nephrology	131 (57.7)
Supportive care	105 (46.3)
Other	16 (7.0)
Area of practice, *n* (%)	
England	152 (67.0)
Northern Ireland	23 (10.1)
Scotland	33 (14.5)
Wales	19 (8.4)
Represented centres within countries in UK, *n* (%)	
All centres	55 (78.6)
England	39 (76.4)
Northern Ireland	5 (100)
Scotland	6 (66.7)
Wales	5 (100)

Abbreviations: % percentage; IQR interquartile range; *n*, number; OT, occupational therapist.

The mean age was 44.6 (SD 10.5) years; and 77.5% (*n* = 176) were female; 81.9% (*n* = 186) were of White ethnicity and 67% (*n* = 152) were in England, 14.5% (*n* = 33) in Scotland, 10.1% (*n* = 23) in NI and 8.4% (*n* = 19) in Wales. The clinical roles of the participants included dietitians (33.9%, *n* = 77), consultant nephrologists (22%, *n* = 50), nurses (HD/transplant) (21.6%, *n* = 49) and consultant transplant surgeons (6.2%, *n* = 14; Table 1) with participants supporting multiple patient groups, with the majority supporting patients on HD (91.6%, *n* = 208).

**FIGURE 1:**
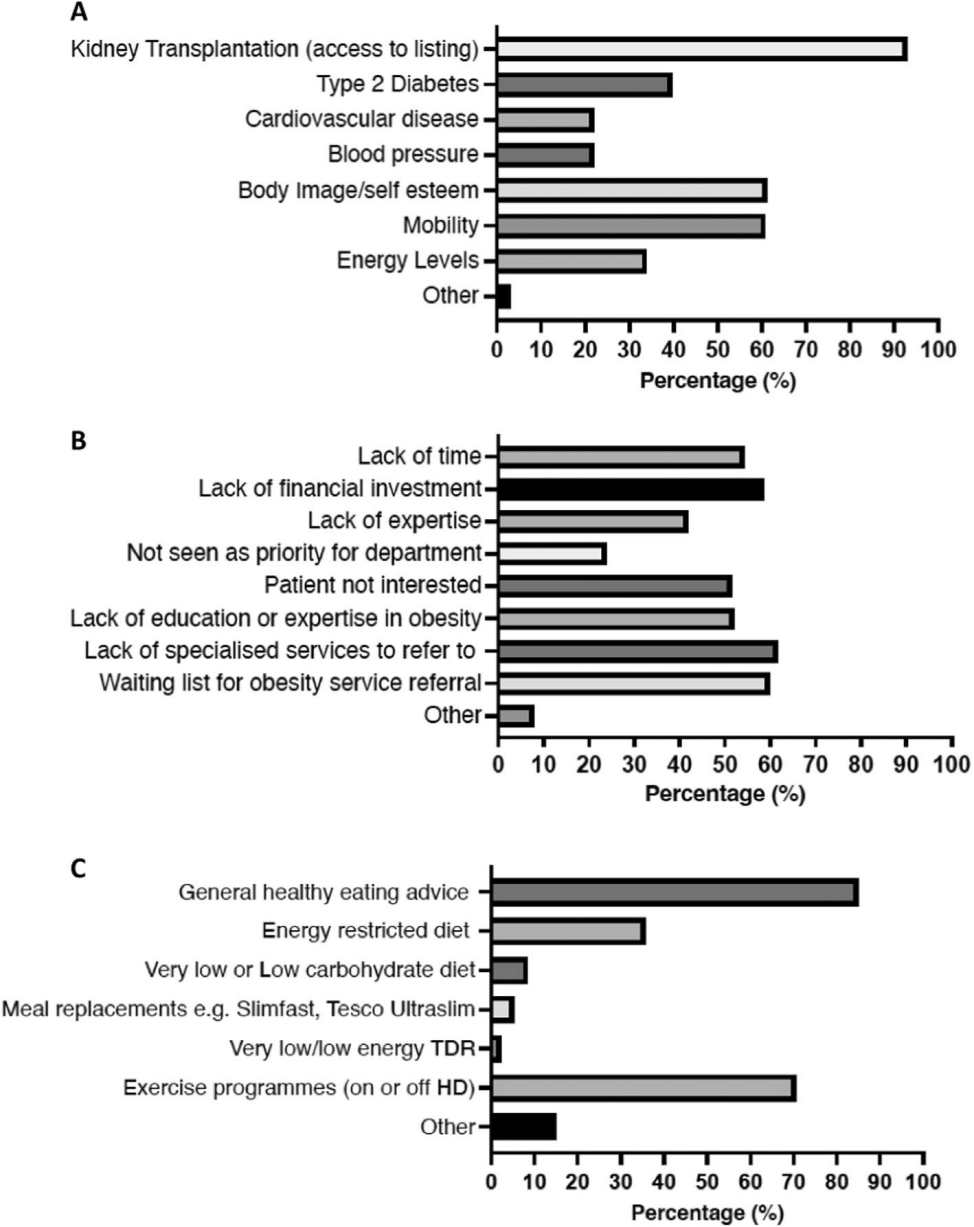
Healthcare providers responses to: (A) challenges in helping patients lose weight with their kidney service; (B) reasons for wishing to lose weight and (C) lifestyle advice given to patients with obesity and KF on haemodialysis to support weight reduction.

Based on their experiences supporting PLwO and KF, 94.7% of participants reported that PLwO on HD expressed a wish to lose weight, with access to KTx the main reason (93%, *n* = 211) followed by self‐esteem/body image (61.2%, *n* = 139) and improved mobility (60.8%, *n* = 138; Figure 1A; Table S5). When asked what are the challenges to HCPs helping patients to lose weight, lack of specialised obesity service to refer to (61.7%, *n* = 140), long waiting lists for obesity management services (59.9%; *n* = 136) and lack of local funding/investment in obesity treatment and support (58.6%, *n* = 133; Figure 1B; Table S5) were most common. Lack of education/expertise within obesity management, patients perceived as not being interested and lack of time were also major barriers to the provision of obesity treament (52%; 51.5% and 54.2%, respectively; see Figure 1B; Table S5).

A total of 60.1% (*n* = 131) of participants reported having access to an obesity service to refer patients to, of these participants, 55% (*n* = 72) reporting local access to a weight management service as part of their kidney service, while 87% (*n* = 114) reporting an obesity service within/outside their hospital centre (Table S6). Individual weight management support was the most common service type available to refer to (40.5% [*n* = 92]; Table S6). Weight loss treatment focussed on healthy eating (85%, *n* = 193) and increasing physical activity (70.5%, *n* = 160; Figure 1C), with dietitians (90.3%, *n* = 205) being the main HCPs supporting this care (Table S7). A total of 38.9% (*n* = 84) reported referring patients to bariatric surgery services and 34.7% (*n* = 75) had a patient on incretin‐based therapies (Table S7). When looking at nephrologists and consultant surgeons (*n* = 64), 57.8% reported referring for bariatric surgery and 51.6% had a patient on an incretin‐based therapies. When asked how many people each participant had referred to bariatric surgery in the preceding 12 months, the median number was 1.0 (IQR 0.0, 2.0) with none reported to have either received bariatric surgery or been listed for transplantation or had undergone a transplant following bariatric surgery (Table S7).

**FIGURE 2:**
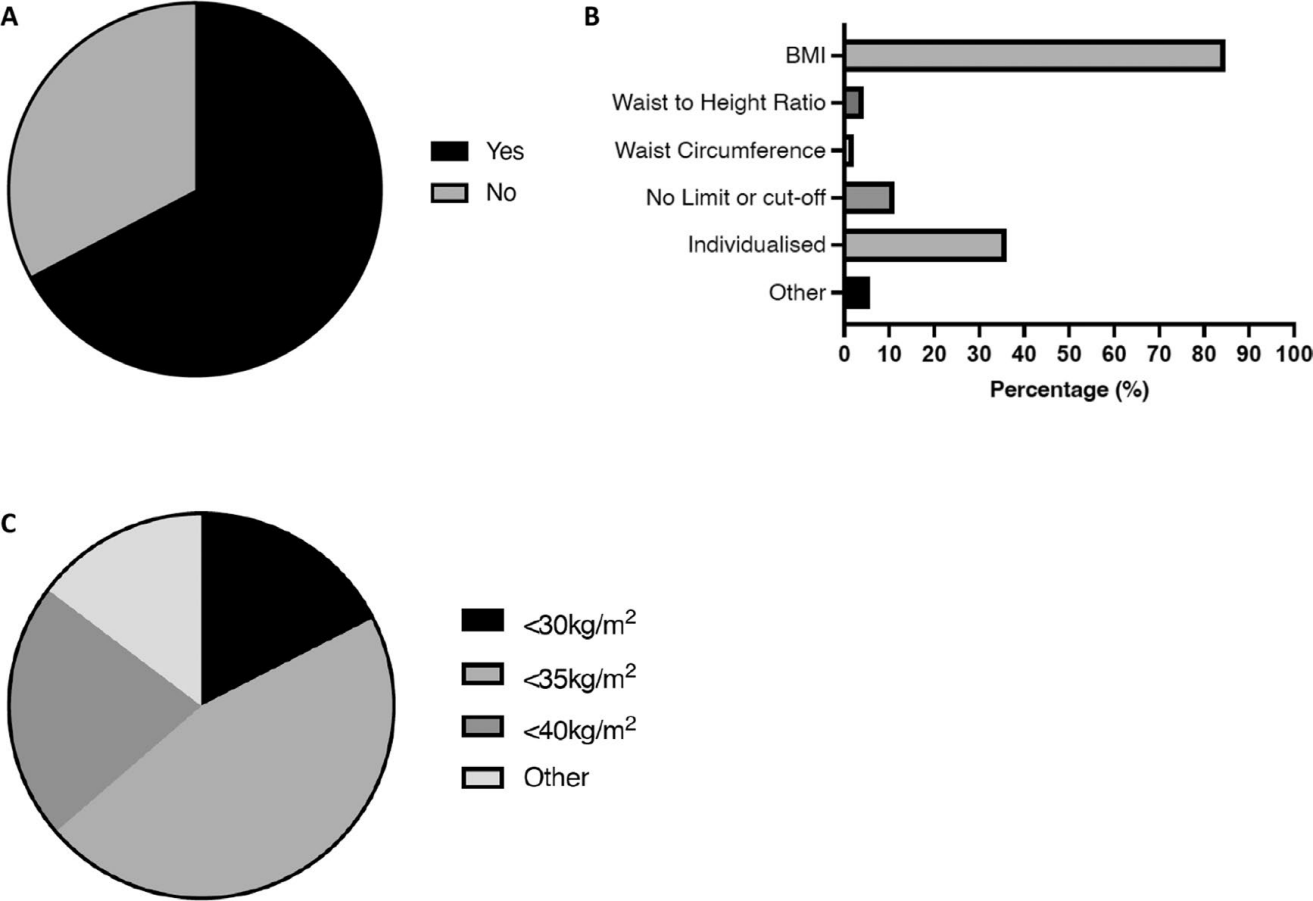
Kidney transplantation referral listing criteria for people living with obesity and KF on haemodialysis. (A) Number of healthcare providers that know the referral listing criteria at your centre or centre you refer to. (B) Referral listing criteria used within the transplant centre. (C) BMI cut‐offs used at centres. BMI, body mass index.

When asked about weight‐related criteria to list patients for KTx at their centre, 67.3% (*n* = 144) reported knowing what these local criteria were (Figure 2A; Table S8). Of those who reported they knew the criteria, the majority reported either body mass index (BMI, 84.7%; *n* = 122) or individualising the criteria to the patient (36.1%, *n* = 52), with 11.1% (*n* = 16) having no BMI criteria (Figure 2B; Table S8). Other measures of adiposity were rarely reported to be used as assessment criteria (2.1% for waist circumference [*n* = 3] or 4.2% [*n* = 6] for waist to height ratio; Figure 2B; Table S8). BMI listing criteria varied significantly across the United Kingdom, with a BMI ≤ 35 kg/m^2^ (54.1%) being the most common cut‐off (Figure 2C; Table S8).

When asked, do the KT listing criteria differ within their transplant centre (e.g., between different surgeons) nearly a quarter (24.5%, *n* = 52) perceived there were differences, with the majority reporting differences in BMI cut‐offs (71.2%, *n* = 37) and individualised surgical criteria (71.2%, *n* = 37; Table S8). Furthermore, only 17.8% (*n* = 38) reported knowing the percentage of patients otherwise medically fit for KTx but not listed because of obesity/elevated BMI.

### 3.1 Comparison of Devolved Nations, Clinical Roles and Transplant Centres Compared With Referral Centres

Summary tables for the comparisons between the devolved nations, centre type and clinical role are in Tables S9 and S10, S11 and S12 and S13 and S14, respectively. When comparing different UK countries, there were differences in challenges in helping patients lose weight, particularly lack of specialist services to refer to (*p* = 0.011) and waiting list for these services (*p* = 0.022); access to obesity management services to help people with KF lose weight (*p* = 0.001); what services were available to refer to (*p* < 0.001); what healthcare professional were involved in supporting patients to lose weight (*p* = 0.003); referral to bariatric surgery (*p* = 0.012) and the use of incretin‐based therapies (*p* < 0.001). No differences were found between transplant/referral centre or clinical roles.

### 3.2 Weight Stigma in Kidney Services

The mean F‐Scale score was 3.42 (SD 0.46) indicating participants reported negative weight bias attitudes towards PLwO (Table 2). Nearly all participants (99.5%; *n* = 206) reported negative attitudes towards PLwO (F‐Scale > 2.5). When assessing fat phobia categories, the majority reported mild fat phobia (63.3% [*n* = 131; F‐Scale 2.51–3.45]), with over a third (35.7% [*n* = 74]) having moderate fat phobia (31.4% [*n* = 65; F‐Scale 3.46–4.39]) or high fat phobia (4.3% [*n* = 9; F‐Scale ≥ 4.4]), and only two participants indicating neutral/positive perceptions (1.0%; Table 2). The F‐Scale had a high level of internal consistency, as determined by a Cronbach's *α* of 0.842.

**TABLE 2:** Weight stigma in UK kidney healthcare providers.

Questionnaire (*n* = 207)	
Fat Phobia Scale (mean [SD])	3.42 (0.46)
Attitudes towards people living with obesity, *n* (%)	
Positive attitudes	1 (0.5)
Negative attitudes	206 (99.5)
Categories of fat phobia, *n* (%)	
No fat phobia	2 (1.0)
Mild fat phobia	131 (63.3)
Moderate fat phobia	65 (31.4)
High fat phobia	9 (4.3)

Abbreviations: % percentage; *n*, number; SDs, standard deviations.

When comparing F‐Scale scores between UK countries, clinical roles and transplant/referral centres (Tables S15–S17, respectively), F‐Scale was significantly higher in NI (mean 3.7 [SD 0.6]) compared with England (3.4 [SD 0.5]), Scotland (3.4 [SD 0.3]) and Wales (3.3 [SD 0.4]; *p* = 0.002). While roles and transplant/referral centre were not associated with fat phobia (both *p* = 0.4).

Univariable and multivariable regressions assessed predictors of fat phobia (Tables S18 and S19, respectively). Ethnicity predicted higher fat phobia, with people from minority ethnic groups having a 0.22 (95% CI 0.06–0.38; *p* = 0.007) higher F‐Scale compared to participants with White ethnicity. Additionally, type of role predicted fat phobia (*p* < 0.001), with allied health professionals having lower fat phobia compared with nephrologists (−0.22 F‐Scale 95% CI −0.38 to −0.05). Surgeons, and other professionals tended to have lower fat phobia compared with nephrologists (−0.10 95% CI –0.36 to 0.17; −0.16 95% CI −0.40 to 0.07 F‐Scale, respectively) and kidney nurses were higher (0.18 95% CI 0.00–0.36 F‐Scale), though these were not statistically significant.

## 4 Discussion

This study provides the first national overview of UK practice, with representation of 78.6% of kidney centres, demonstrating inconsistent access to obesity services, a lack of consensus on listing criteria for KTx and that kidney HCPs have weight biased attitudes towards PLwO.

Despite 94.7% of kidney HCPs reporting that patients wished to lose weight, principally to enhance access to KTx, only 60.1% had access to a dedicated referral pathway to specialist obesity services, with substantial variation observed across the United Kingdom. Key barriers to supporting weight loss included limited access to specialist services, long waiting lists and insufficient local funding/investment. These challenges are not confined to kidney services but instead reflect broader regional inequalities across the United Kingdom in provision of specialist obesity services. Access to such services is highly variable with reported availability ranging from 44% to 83% [32, 33, 34]. Despite this, referral rates to specialist weight management and bariatric surgery services remain low despite people being eligible [32]. Furthermore, our data showed some kidney services were offering exemplar support for patients, while others had no capacity or commissioned (publicly funded) services at all (e.g., NI). Challenges in access may also be exacerbated by weight management services excluding patients due to perceived dietary complexities for people living with CKD [26]. These data highlight the challenges kidney services face in supporting people living with obesity and KF who are potentially eligible for KTx to access obesity treatment, and reinforce clinicians' views that, although many patients wish to do so [16], access and referral to services remains limited.

In addition, most obesity services were external to the participants' kidney centre (87%). Concerningly, external referrals are prone to communication breakdowns, fragmentation of care, increasing safety risk and patients disappearing into a ‘referral black hole’ [35, 36]. The lack of local access and reliance on external services risks delaying transplantation, disrupting collaborative care and reducing timely referrals. As a result, PLwO who might otherwise be eligible for KTx can wait substantially longer, potentially losing their pre‐emptive status or experiencing prolonged time on HD, both of which negatively affect patient outcomes and access to KTx [37, 38, 39].

International transplantation guidelines recommended that obesity alone should not preclude KTx [15, 17, 40, 41, 42]; however the use of BMI based criteria remain common [20, 21, 25]. Our data is consistent with previously‐published international data, revealing BMI was consistently used to assess KTx eligibility (84.7%), alongside substantial UK variation of BMI cut‐offs. Given the lack of consensus on when obesity makes perioperative risk or longterm outcomes unacceptable [41], individual centres appear to be creating their own criteria, leading to variation in BMI cut‐offs. Interestingly, the most common UK BMI cut‐off was ≤ 35 kg/m^2^, which is considerably lower than the recently reported US threshold of 40 kg/m^2^ [25]. This difference may reflect a more cautious UK approach, as well as the wide‐spread adoption of robotic KTx, widely used in the United States, which potentially enables transplantation in patients with higher BMI by overcoming some of the traditional challenges of KTx in PLwO [43]. In addition, this inconsistency may also reflect the limitations of BMI as a measure of obesity [18, 44] and KTx risk, particularly its inability to account for fat distribution, highlighting the need for additional measures such as waist circumference and waist‐to‐hip ratio [40, 41]. However, these measures were rarely used in UK practice, mirroring US data and highlighting an area for improvement for clinical practice [25]. Finally it should be acknowledged that, as previously mentioned, the established association between obesity and adverse KTx outcomes [45] may have led to BMI cut‐offs being lower in some kidney centres. Interestingly, HCPs identified that listing criteria differed within their transplant centre (24.5%), with BMI being the main difference. It is unclear what is driving this, though data suggests inconsistency or a lack of local KTx obesity policies maybe partially responsible [20]. In addition, nearly a third of participants, mainly non‐surgical team members, reported not knowing their local weight‐related listing criteria for KTx, suggesting gaps in communication within some kidney services or use of individualised assessments without a one‐size‐fits‐all BMI approach. This variation in practice can negatively impact patients, who report feeling unfairly treated and not understanding why they are not eligible for KTx [16, 24].

One area of agreement across international guidelines is PLwO should lose weight to be eligible for KTx [15, 40, 41, 42]. Obesity management, like CKD, requires a MDT approach to optimise outcomes [17, 18, 31, 46, 47, 48]. From our data, however, UK kidney services lack access to HCPs other than dietitians to support PLwO to lose weight. US data demonstrates access to nutritional counselling alone for weight loss is limited and often ineffective, with only 10%–26.3% of people with a BMI < 35 kg/m^2^ losing weight, just 5% reaching the listing target and none achieving weight loss when BMI > 40 kg/m^2^ [49]. Based on our findings this lack of efficacy may be partially explained by obesity treatment focussing on non‐specific dietary and physical activity advice, without adequate behavioural change support. Such approaches typically result in only modest weight loss and can be resource intensive to deliver [17, 31]. In addition, limited high‐quality evidence regarding effective non‐surgical weight loss interventions in PLwO and KF, particularly those undergoing HD [19] alongside a lack of training in obesity management [16] may leave clinicians unsure how best to support weight loss. This lack of randomised controlled trials in CKD populations is further reflected in international KTx guidelines where no obesity treatment pathway is offered other than bariatric surgery [15, 17, 40, 41, 42]. Despite this, only 39% of UK HCPs reported referring patients for metabolic/bariatric surgery services, with no participants reporting their patients having metabolic/bariatric surgery in the last 12‐months. Poor access to bariatric services, long referral waiting times and lack of time within consultations to support weight loss, are confirmed by other studies [16, 22, 24]. Views on referral for bariatric surgery are mixed, with nephrologists, surgeons and transplant nephrologists generally supportive, while other specialties (e.g., some dietitians) view it as invasive and express concerns about postoperative complications [16, 20, 24]. This is further compounded by kidney dietitians and other HCPs perceiving obesity management to lie outside their scope of practice and lacking clarity about their role in addressing obesity [16].

Despite growing interest in using incretin‐based therapies to optimise PLwO for KTx eligibility [17, 18], even fewer participants (34.7%) had patients using them. The low usage of these therapies may reflect a lack of evidence for their use in patients undergoing HD or prior to transplantation, specifically for obesity [18, 50, 51] and limited clinician exposure and experience [16], due to UK specific restrictions on prescribing these medications within the NHS [52] and KF is not an approved indication for prescription [53]. Furthermore, both our findings and previous studies [16, 22, 24] highlight gaps in obesity management education/expertise, limited time to support weight loss, competing clinical priorities and uncertainty among clinicians about whether weight management is part of their role. Together, these factors may undermine clinicians' confidence and capacity in delivering obesity treatment and safely escalating care. With the recently published American Society of Nephrology guidelines [17] and conclusions from the KDIGO controversies conference on obesity [18] offering clearer guidance on obesity management in CKD, this may support improved services offered to PLwO in UK clinical services. However, successful implementation remains challenging without a skilled workforce and structures to support obesity treatment as a chronic condition.

Weight stigma is highly prevalent within society including in healthcare [54, 55], affecting both physical and psychological health and impacting equable access to healthcare services [54], and was recently highlighted internationally [17]. Our study is the first to measure weight stigma in kidney HCPs and showed nearly all participants had negative attitudes towards PLwO based on the F‐Scale and over a third have moderate/high fat phobia. The mean F‐Scale in our sample was similar to pooled data on weight bias in different healthcare professionals [56]. These negative attitudes are evident in participants' responses, with over half of HCPs reporting patients were not interested in losing weight, despite nearly 95% reporting that patients wished to lose weight. These opinions may reflect wider societal weight bias that PLwO lack self‐discipline, are weak‐willed and lazy [54, 55], and may be compounded by previous negative surgical experiences, such as postoperative KTx complications, that are attributed to patients' weight, reinforcing negative stereotypes. This raises the possibility that weight stigma may affect equitable access to obesity services and KTx, as well as timely and appropriate referrals for weight‐loss support. To address these issues there is a need for a cohesive approach towards managing obesity, with cross organisation collaboration supported by appropriate funding to overcome local and system barriers. See Table 3 for suggested recommendations.

**TABLE 3:** Recommendations for improving obesity management and transplant access in the United Kingdom [16, 17, 18].

Recommendation area	Specific recommendations
National policy and guidelines	Develop nationally agreed UK guidelines on obesity management and transplant listing criteria. Standardise BMI thresholds and listing practices. Establish consistent national criteria for equitable access.
Evidence generation	Invest in targeted funding and research infrastructure to support well‐designed, adequately powered studies.
Training and workforce development	Implement national training on obesity as a chronic disease, weight‐loss interventions including incretin therapies, reducing stigma and awareness of specialist services.
Service provision and pathway development	Expand specialist obesity services and improve access to structured weight‐management pathways.
Multidisciplinary care priority	Develop more tailored multidisciplinary care to support PLwO to lose weight, making obesity a greater priority within kidney services and recognising it as a key treatment outcome that improves equity to KTx and enhances patient quality of life.
Centres of excellence	Establish centralised centres for higher‐BMI transplant candidates to reduce variation and improve access.
Multidisciplinary collaboration	Strengthen communication between kidney services and transplant surgeons to ensure clear listing criteria.
Equity and access	Ensure pathway improvements address service gaps and promote equitable access for all PLwO seeking transplantation.

Abbreviations: KTx, kidney transplant; PLwO, patient living with obesity.

This study has several strengths; it is the largest UK survey to date and offers an overview of the current state of access, service provision and KTx listing criteria, offering a starting place to address the barriers to access for PLwO and KF on HD. There was excellent national representation, with 78.6% of UK centres participating, supporting generalisability within the United Kingdom. However, as the study was conducted exclusively in the United Kingdom, the findings may not fully translate to international settings. Furthermore, responses were collected across multiple centres, with three centres contributed disproportionately, which may introduce centre‐specific bias and should be considered when further interpreting generalisability. In addition, a range of different professional views were gathered, an issue found from previous studies [20, 21, 22, 23, 24], therefore enabling a widerpplicability across healthcare professions. Nevertheless, the relatively low involvement of transplant surgeons and psychologists means their views may not be underrepresented. Given that transplant surgeons ultimately determine KT eligibility, their limited participation introduces the potential for reporting bias and should be acknowledged. There are also limitations to the study that should be considered when interpreting the results. The study was undertaken at a single time‐point, and in this rapidly changing area, attitudes and practice may have changed by the time of publication. The participants were mainly female and of White ethnicity and therefore may not represent that opinions of all healthcare providers. Patients on peritoneal dialysis may also face challenges but were not considered in this analysis. The use of purposive and snowball sampling may have introduced selection bias, as HCPs with a greater interest in obesity management may have been more likely to participate. However, this approach also helped capture views from wide range of HCPs and those regularly involved in this area, offering a more relevant reflection of current practice.

## 5 Conclusion

Within the United Kingdom, there is limited access to obesity management services for PLwO and KF on HD prior to KTx. This appears to be driven by a combination of organisational challenges such as inadequate funding and individual‐level barriers including weight stigma and lack of specialist obesity expertise. While some exemplar services exist, offering comprehensive MDT care, others offer minimal support to PLwO resulting in significant variation in patient experience and potential outcomes. Inconsistent transplant listing criteria both between and within services has the potential to directly impact access to KTx potentially creating a post‐code lottery in care. Our findings highlight there is an urgent need for a paradigm shift in the way obesity is managed within UK kidney and wider health services. Only through collaborative action can comprehensive obesity treatment pathways be established that meet the needs of this vulnerable patient population.

## Author Contributions

A.B. conceived the study and was the senior author. A.B., K.M., H.L.M., S.G., S.P., R.L.B. and R.M. contributed to the study and survey design and methodology. A.B. was responsible for the oversight of the study. A.B., K.M., H.L.M., S.G., S.P., R.T. and R.M. contributed to the recruitment of participants. A.B. and V.V. were responsible for the data analysis. All authors contributed to data interpretation, and the writing of the manuscript. All authors contributed to critical revision of the manuscript and gave final approval.

## Funding

A.B. is funded by the National Institute for Health and Care Research with an Advanced Fellowship (NIHR303041). The funder was not involved in the design and conduct of the review, analysis and interpretation of the data; preparation, review or approval of the manuscript; and decision to submit the manuscript for publication.

## Conflicts of Interest

A.B. declares researcher‐led grants from the National Institute for Health Research, Rosetrees Trust, British Dietetic Association, British Association of Parenteral and Enteral Nutrition and BBRSC, A.B. has received travel a travel grant from Chiesi Farmaceutici for conference attendance. A.B. reports honoraria from Novo Nordisk, Nutricia, Danone, Mac Nutrition and Eli Lilly outside the submitted work and is on the Medical Advisory Board and shareholder of Reset Health Clinics Ltd. R.L.B. declares researcher‐led grants from the National Institute for Health Research, Rosetrees Trust, Sir Jules Thorn Biomedical Trust and NovoNordisk. R.L.B. reports honoraria from Novo Nordisk, Eli Lilly, Medscape, ViiV Healthcare and International Medical Press and advisory board and consultancy work for Novo Nordisk, Eli Lilly, Pfizer, Gila Therapeutics, Epitomee Medical and ViiV Healthcare and from May 2023 is an employee and shareholder of Eli Lilly and Company. H.L.M. declare researcher‐led grants from Queensland Health Office of the Chief Allied Health Officer 2023–2025. H.L.M. reports support from KDIGO supporting travel to attend conference and is Deputy Chair Chronic Kidney Disease Work Group: Australasian Clinical Trials Network. R.M. declares research‐led grants from the National Institute for Health Research, Kidney Research UK, Rosetress Trust and Royal Free Chrity. S.G. is director of Kidney Beam Ltd. The other authors declare no conflicts of interest.

## Supporting information


**Table S1:** Represented kidney centres in England.
**Table S2:** Represented kidney centres in Northern Ireland.
**Table S3:** Represented kidney centres in Scotland.
**Table S4:** Represented kidney centres in Wales.
**Table S5:** Healthcare providers responses to challenges in helping patients lose weight with their kidney services and reasons for wishing to lose weight.
**Table S6:** Access to obesity services.
**Table S7:** What is offered in obesity management service.
**Table S8:** Kidney transplantation listing referral criteria.
**Table S9:** Demographics about participants by devolved nations (*n* = 227).
**Table S10:** Survey questions by devolved nations (*n* = 227).
**Table S11:** Demographics about participants by clinical role (*n* = 227).
**Table S12:** Survey questions by clinical roles (*n* = 227).
**Table S13:** Demographics about participants comparing transplant vs. referral centres (*n* = 227).
**Table S14:** Survey questions comparing transplant versus referral centres (*n* = 227).
**Table S15:** F‐Scale for participant by devolved nations (*n* = 210).
**Table S16:** F‐Scale for participant by clinical role (*n* = 210).
**Table S17:** F‐Scale comparing transplant versus referral centres (*n* = 210).
**Table S18:** Univariable regression outputs, exploring the predictors of the Fat Phobia Scale.
**Table S19:** Multivariable regression outputs, exploring the predictors of the Fat Phobia Scale.

## Data Availability

De‐identified participant data that underlie the results reported in this article will be made available on publication and ending 5 years after publication. Proposals should be made to the corresponding author and will require a data access agreement.
