# Supplementary material for: Supporting Weight Management for Kidney Transplant Candidates in People With Obesity on Haemodialysis: The First National Survey of UK Kidney Healthcare Providers
Source: Diabetes Obes Metab. 2026 May 31;28(8):7116–25. doi: 10.1111/dom.70900 (PMC13341359; doi:10.1111/dom.70900)
Supplement: Supplementary file 1 — Table S1: Represented kidney centres in England. Table S2: Represented kidney centres in Northern Ireland. Table S3: Represented kidney centres in Scotland. Table S4: Represented kidney centres in Wales. Table S5: Healthcare providers responses to challenges in helping patients lose weight with their kidney services and reasons for wishing to lose weight. Table S6: Access to obesity services. Table S7: What is offered in obesity management service. Table S8: Kidney transplantation listing referral criteria. Table S9: Demographics about participants by devolved nations (n = 227). Table S10: Survey questions by devolved nations (n = 227). Table S11: Demographics about participants by clinical role (n = 227). Table S12: Survey questions by clinical roles (n = 227). Table S13: Demographics about participants comparing transplant vs. referral centres (n = 227). Table S14: Survey questions comparing transplant versus referral centres (n = 227). Table S15: F‐Scale for participant by devolved nations (n = 210). Table S16: F‐Scale for participant by clinical role (n = 210). Table S17: F‐Scale comparing transplant versus referral centres (n = 210). Table S18: Univariable regression outputs, exploring the predictors of the Fat Phobia Scale. Table S19: Multivariable regression outputs, exploring the predictors of the Fat Phobia Scale. [file DOM-28-7116-s001.docx]

**Supplementary Material**

**National Survey**

**Screening question**

- Do you encounter or care for patients living with obesity and kidney failure (CKD stage 5) on heamodialysis within your practice? Yes/No

**Block 1: Demographics**

This section will collection basic information including about your role, gender, ethnicity, patients you support and where you work in the UK.

- What is your role within the service? Dietitian; Nephrologist; Psychologist; Nurse; Surgeon, Social Worker, Other, please state
- Age – 18-24; 25-29; 30-34; 35-39; 40-44; 45-49; 50-54; 55-59; 60-64; 65-69; 70+
- Gender 1) male 2) female 3) transgender 4) other 5) prefer not to say
- Ethnicity 1) White 2) Mixed/Multiple Ethnic groups 3) Asian 4) Black 5) Other ethnic group
- Country of work – 1) England 2) Scotland 3) Wales 4) Northern Ireland
- Which patients do you support in your professional role? (tick all that apply) Those undergoing – 1) haemodialysis 2) PD 3) transplant 4) advanced kidney care e.g., low clearance clinics 5) general nephrology 6) supportive care 7) Other please specify
- How many years’ experience do you have working with patient with kidney disease (years, and months)? Updated options
- Which UK kidney centre do you work in?.
  - England – list all renal and transplant units in England
  - Wales – list all renal and transplant units in Wales
  - Scotland – list all renal and transplant units in Scotland
  - NI – list all renal and transplant units in NI

**Block 2: Understanding what is offered to people living with obesity and kidney failure (CKD stage 5) on haemodialysis within units**

This section will look at the challenges of help people living with obesity and kidney failure and what your service offers patients at the present time.

**Service logistics**

- Have any patients with obesity and kidney failure on heamodialysis ever expressed that they would like to lose weight? Yes/No, If yes give reasons:
- Kidney transplantation
- Type 2 diabetes
- Cardiovascular disease
- Blood pressure
- Body Image/self esteem
- Mobility
- Energy levels
- Other please specify
- What are the challenges to helping these patients lose weight?
- Lack of time
- Lack of financial investment/resources
- Lack of expertise
- Not seen as a priority in your department
- Patients not interested
- Lack of education or expertise within the area of obesity management
- Lack of specialised services to refer to
- Waiting list for obesity service referral
- Other - Please specify in box (free text)
- What gaps do you see in the provision of weight management services for patients with CKD living with obesity in your area?

**Services offered to patients**

- Do you have access to a service to help people with kidney failure (CKD stage 5) to lose weight? Yes/No
  - Yes: within your renal service
  - Yes: referral to weight management services within or outside your trust
- Please tick what services are available to patients to be referred to (each question then offer free text to describe what is offered, e.g., number of session, where, follow up)
  - Individual weight management support
  - Group weight management programme
  - Pharmacotherapy support
  - Bariatric surgery
  - Other (please state)
  - If no, what has prevented you from offering weight management support? Please explain (free text)
- What lifestyle advice do you give patients with obesity and kidney failure on heamodialysis to support weight reduction?
  - General healthy eating advice
  - Energy restricted diet
  - Very low or Low carbohydrate
  - Meal replacements e.g. Slimfast, Tesco Ultraslim
  - Very low/Low meal replacement products
  - Exercise/physical activity programmes, on and off heamodialysis, e.g., Kidney Beam or referral to physiotherapist
  - Other: Please specify in box (free text)
- Which healthcare professionals are involved with helping patients with obesity and kidney failure on heamodialysis to lose weight? (tick all that apply)
  - Specialist Dietitian e.g. renal or weight management
  - Specialist Psychologist e.g., clinical psychologist, counsellor
  - Specialist Weight management consultant physician/Endocrinologist
  - Specialist Physical Activity Specialist e.g., Physiotherapy
  - Other (please specify)
- Each question will then open up and ask were
  - If yes, what do you offer?
- Have you referred any patients to bariatric surgery prior to kidney transplant listing? Yes/No
- If yes, please give
  - How many patients have you referred for bariatric surgery in past 12 months?
  - How many received surgery?
  - How many were listed?
  - How many received a transplant?
  - If not, what has stopped you referring them to bariatric surgery
- Do you have any patients who have started GLP-1 receptor agonists e.g., Liraglutide, Semaglutide or GLP/GIP receptor agonists e.g. Tazepetide? Yes/No
  - If yes, what medication were they offering?
  - If not, what has stopped you offering GLP-1/GIP RA

**Block 3: Referral Criteria**

- Do you know the referral criteria at your centre, if any, for listing patients for kidney transplantation in those living with obesity and kidney failure? (e.g., BMI, waist to height ratio or waist circumference)? Yes/No

If yes, ask following questions:

- Do you use BMI and/or waist circumference and/or waist to height ratio within the criteria?
- BMI
- Waist to Height ratio
- Waist Circumference
- There is no limit or cut-off
- Individualised
- If BMI –
  - BMI<30kg/m^2^;
  - BMI<35kg/m^2^;
  - BMI<40kg/m^2^
  - Other (please state)
- Waist to height ratio -
  - ≥0.6
  - ≥0.7
  - ≥0.8
  - Other please state
- If yes, what are the reasons for using this/these?
- Waist Circumference
  - <94cm (men)
  - <108cm (men)
  - <80cm (women)
  - <88cm (women)
  - Other please state
- Does your referral criterion differ if a person is also living with diabetes? Yes/No
- If yes, can you please explain how?
- Does the referral criterion differ within your transplanting centre? E.g., between different surgeons. Yes/No/Don’t know
  - If yes, what are the differences?
    - BMI
    - Waist to Height ratio
    - Individual surgical assessment
    - Other (please state)
- Do you have a separate transplant assessment pathway for people with obesity and kidney failure? If so, please give details of how this differs.
- Do you know how many people who are medically fit for kidney transplant not listed as a result of obesity/elevated BMI? Yes/No
  - If yes, please can you specify/estimate how many in percent (%) (free text)
- Do you have access to a service to help kidney donors to lose weight?
  - If yes, what do you offer?

**Block 4: Final comments**

- Any other comments on your experiences of supporting patients with obesity and kidney failure on heamodialysis to lose weight which are not covered within the survey?

**Block 5: Fat Phobia Scale (validated questionnaire)**

This is a validated questionnaire to help understand healthcare professional views of people living with obesity.

Listed below are 14 pairs of adjectives sometime used to describe people living with obesity. For each adjective pair, please mark in the box closest to the adjective that you feel best describes your feelings and beliefs about people living with obesity.

| 1 | Lazy |  |  |  |  |  | Industrious |
| --- | --- | --- | --- | --- | --- | --- | --- |
| 2 | No will power |  |  |  |  |  | Has will power |
| 3 | attractive |  |  |  |  |  | Unattractive |
| 4 | Good self-control |  |  |  |  |  | Poor self-control |
| 5 | fast |  |  |  |  |  | Slow |
| 6 | Having endurance |  |  |  |  |  | Having no endurance |
| 7 | active |  |  |  |  |  | Inactive |
| 8 | Weak |  |  |  |  |  | strong |
| 9 | Self-indulgent |  |  |  |  |  | Self-sacrificing |
| 10 | Dislikes food |  |  |  |  |  | Likes food |
| 11 | shapeless |  |  |  |  |  | Shapely |
| 12 | Undereats |  |  |  |  |  | Overeats |
| 13 | Insecure |  |  |  |  |  | Secure |
| 14 | Low self-esteem |  |  |  |  |  | High self-esteem |

**Survey Development**

The survey was developed in collaboration with 18 UK kidney experts including nephrologists, transplant surgeons, clinical psychologists, allied health professionals and patient advocates alongside previous research ^20^. Prior to dissemination the survey was piloted and adjustments made according to feedback, this ensured that the length, terminology and questions were targeted and appropriate to the study aims. Robotic kidney transplantation is increasingly used worldwide for patients with a high body mass index (BMI) but it is not routinely performed in the UK as such it excluded from our survey.

**Sample Size**

There were 70 kidney centres in the UK identified from the Renal Registry, serving approximately 71,000 patients on renal replacement therapy (representing a prevalence of 1323 per million population), of which about 38% receive haemodialysis ^1^. Following discussion with the study statistician (VV) and review of previous published surveys of the prevalence of obesity and kidney failure ^2^, it was estimated that a reply rate of 50% from kidney centres would be considered a good response, giving approximately 35 kidney centres replying. It was estimated that on average 3 members of staff would complete from each provider to give a total of 105 participants. The age range (18-70) was representative of the healthcare providers being surveyed. Multiple members of staff at each kidney centre were able to complete the survey to allow a broad range of responses and perspectives of different professions.

**Table S1 Represented Kidney Centres in England**

| **Which UK kidney centre do you work in England? *n* (%)** | **(n=152)** |
| --- | --- |
| Birmingham Heartlands Hospital and Queen Elizabeth Hospital | 5 (3.3) |
| Basildon Hospital | 4 (2.6) |
| Brighton Royal Sussex County Hospital | 4 (2.6) |
| Bristol Southmead Hospital | 4 (2.6) |
| Cambridge Addenbrookes Hospital | 6 (3.9) |
| Carlisle Cumberland Infirmary | 1 (0.7) |
| Carshalton St Helier Hospital | 4 (2.6) |
| Coventry University Hospital Coventry and Warwick | 1 (0.7) |
| Derby Royal Derby Hospital | 2 (1.3) |
| Dorchester Dorset County Hospital | 1 (0.7) |
| Dudley Russells Hall Hospital | 1 (0.7) |
| Exeter Royal Devon and Exter Hospital | 1 (0.7) |
| Gloucestershire Royal Hospital | 2 (1.3) |
| Hull Royal Infirmary | 3 (2.0) |
| Ipswich Ipswich Hospital | 1 (0.7) |
| Kent and Canterbury Hospital | 5 (3.3) |
| London St Bartholomews Hospital and The Royal London Hospital | 3 (2.0) |
| London Guys Hospital and St Thomas Hospital | 9 (5.9) |
| London Kings College Hospital | 6 (3.9) |
| London Royal Free, Middlesex and UCL Hospitals | 11 (7.2) |
| London St Georges Hospital and Queen Marys Hospital | 2 (1.3) |
| London Hammersmith, Charing Cross and St Marys Hospitals | 3 (2.0) |
| Leeds St James University Hospital and Leeds General Infirmary | 1 (0.7) |
| Leicester General Hospital | 6 (3.9) |
| Liverpool Royal Liverpool University Hospital | 4 (2.6) |
| Manchester Royal Infirmary | 2 (1.3) |
| Newcastle Freeman Hospital and Royal Victoria Infirmary | 6 (3.9) |
| Norfolk and Norwich University Hospital | 2 (1.3) |
| Nottingham City Hospital | 5 (3.3) |
| Oxford Radcliffe Hospital | 7 (4.6) |
| Portsmouth Queen Alexandra Hospital | 14 (9.2) |
| Salford Royal Hospital | 1 (0.7) |
| Sheffield Northern General Hospital | 2 (1.3) |
| Stevenage Lister Hospital | 13 (8.6) |
| Stoke University Hospital of North Staffordshire | 4 (2.6) |
| Sunderland Royal Hospital | 2 (1.3) |
| Wirral Birkenhead Arrowe Park Hospital | 2 (1.3) |
| York District General Hospital | 1 (0.7) |
| Other | 1 (0.7) |
| **Number of England kidney centres represented** | 39 (76.4) |

**Table S2 Represented Kidney Centres in Northern Ireland**

| **Which UK kidney centre do you work in Northern Ireland? *n* (%)** | **(n=23)** |
| --- | --- |
| Antrim Hospital (Northern Trust) | 3 (13.0) |
| Belfast City Hospital | 7 (30.4) |
| Newry Daisy Hill Hospital (Southern Trust) | 7 (30.4) |
| Belfast Ulster Hospital | 5 (21.7) |
| Londonderry and Omagh Tyrone County Hospital (Western Trust) | 1 (4.3) |
| **Number of Northern Ireland kidney centres represented** | 5 (100) |

**Table S3 Represented Kidney Centres in Scotland**

| **Which UK kidney centre do you work in Scotland? *n* (%)** | **(n=33)** |
| --- | --- |
| Airdrie University Hospital Monklands | 3 (9.1) |
| Dundee Ninewells Hospital | 1 (3.0) |
| Edinburgh Royal Infirmary of Edinburgh | 14 (42.4) |
| Glasgow Queen Elizabeth University Hospital | 12 (36.4) |
| Inverness Raigmore Hospital | 2 (6.1) |
| Kirkcaldy Victoria Hospital | 1 (3.0) |
| **Number of Scotland kidney centres represented** | 6 (66.7) |

**Table S4 Represented Kidney Centres in Wales**

| **Which UK kidney centre do you work in Wales? *n* (%)** | **(n=19)** |
| --- | --- |
| Bangor Ysbyty Gwynedd | 2 (10.5) |
| Cardiff University Hospital of Wales | 9 (47.4) |
| Clwyd Ysbyty Glan Clwyd Hospital | 1 (5.3) |
| Swansea Morriston Hospital | 4 (21.1) |
| Wrexham Maelor Hospital | 3 (15.8) |
| **Number of Wales kidney centres represented** | 5 (100) |

**Table S5 Healthcare providers responses to challenges in helping patients lose weight with their kidney services and reasons for wishing to lose weight.**

| **Questions** | ***n* (%)** |
| --- | --- |
| **Have any patients with obesity and kidney failure on haemodialysis ever expressed wishing to lose weight to you? (n=227)** |  |
| Yes | 215 (94.7) |
| No | 12 (5.3) |
| **What reasons were given by the patients for wanting to lose weight? (n=227)** |  |
| Kidney Transplantation [access to kidney transplantation listing] | 211 (93.0) |
| Type 2 diabetes | 90 (39.6) |
| Cardiovascular disease | 50 (22.0) |
| Blood pressure | 50 (22.0) |
| Body image/self esteem | 139 (61.2) |
| Mobility | 138 (60.8) |
| Energy levels | 77 (33.9) |
| Other | 7 (3.1) |
| **What are the challenges to helping these patients lose weight? (n=227)** |  |
| Lack of time | 123 (54.2) |
| Lack of financial investment/resources | 133 (58.6) |
| Lack of expertise | 95 (41.9) |
| Not seen as a priority in your department | 54 (23.8) |
| Patients not interested | 117 (51.5) |
| Lack of education or expertise within the area of obesity management | 118 (52.0) |
| Lack of specialised services to refer to | 140 (61.7) |
| Waiting list for obesity service referral | 136 (59.9) |
| Other | 18 (7.9) |

N, number; % percentage

**Table S6 Access to obesity services**

| **Questions** | ***n* (%)** |
| --- | --- |
| **Do you have access to an obesity management service to help people with kidney failure lose weight? (CKD stage 5) (n=218)** |  |
| Yes | 131 (60.1) |
| No | 57 (26.1) |
| Don’t know | 30 (13.8) |
| **Obesity service within your kidney service? (n=131)** |  |
| Yes | 72 (55.0) |
| No | 59 (45.0) |
| **Referral to specialist obesity service within or outside your trust (n=131)** |  |
| Yes | 114 (87.0) |
| No | 17 (13.0) |

N, number; % percentage; CKD, chronic kidney disease

**Table S7 What is offered in obesity management service**

**This section will look at what services there are available to people living with obesity and kidney failure on haemodialysis**

| **Questions** | ***n* (%)** |
| --- | --- |
| **What services are available for patients to be referred to? (n=227)** |  |
| Individual weight management support | 92 (40.5) |
| Group weight management program | 61 (26.9) |
| Pharmacotherapy support | 36 (15.9) |
| Bariatric surgery | 69 (30.4) |
| Other | 13 (5.7) |
| **What lifestyle advice do you give patients with obesity and kidney failure on haemodialysis to support weight reduction? (n=227)** |  |
| General healthy eating advice | 193 (85.0) |
| Energy restricted diet | 81 (35.7) |
| Very low or Low carbohydrate diet | 19 (8.4) |
| Meal replacements e.g. Slimfast, Tesco Ultraslim | 12 (5.3) |
| Very low/Low [energy] total diet replacement products | 5 (2.2) |
| Exercise programmes, on or off haemodialysis | 160 (70.5) |
| Other | 34 (15.0) |
| **Which healthcare professionals are involved with helping patients with obesity and kidney disease to lose weight (n=227)** |  |
| Specialist Dietitian e.g. renal or weight management | 205 (90.3) |
| Specialist Psychologist e.g., clinical psychologist, counsellor | 60 (26.4) |
| Specialist Weight management consultant physician/Endocrinologist | 19 (8.4) |
| Specialist Physical Activity Specialist e.g. Physiotherapy | 35 (15.4) |
| Other | 23 (10.1) |
| **Do you have any patients who have started GLP-1 receptor agonists e.g. Liraglutide, Semaglutide or GLP/GIP receptor agonists [e.g Tirzepatide; incretin-based therapies]? (n=216)** |  |
| Yes | 75 (34.7) |
| No | 51 (23.6) |
| Don’t know | 90 (41.7) |
| **Have you referred any patients to bariatric surgery prior to kidney transplant to help them lose weight? (n=216)** |  |
| Yes | 84 (38.9) |
| No | 132 (61.1) |
| **How many patients have you referred to bariatric surgery in the last 12 months (n=67) (median [IQR])** | 1.0 (0.0, 2.0) |
| **How many patients have undergone bariatric surgery in the last 12 months (n=58) (median [IQR])** | 0.0 (0.0, 1.0) |
| **How many patients have been listed following undergoing bariatric surgery in the last 12 months (n=32) (median [IQR])** | 0.0 (0.0, 1.0) |
| **How many patients have had a kidney transplant following undergoing bariatric surgery in the last 12 months (n=29) (median [IQR])** | 0.0 (0.0, 0.5) |

N, number; % percentage; BMI, body mass index; IQR, interquartile range; GLP-1, glucagon like peptide-1; GIP, glucose insulinotropic polypeptide

**Table S8 Kidney transplantation listing referral criteria**

**This section will look at the kidney transplantation referral criteria for people living with obesity and KF on haemodialysis**

| **Questions** | ***n* (%)** |
| --- | --- |
| **Do you know the referral criteria at your centre or centre you refer to, if any, for listing patients for kidney transplantation in patients with obesity and kidney failure (e.g. BMI, waist to height ratio, or waist circumference) (n=214)** |  |
| Yes | 144 (67.3) |
| No | 70 (32.7) |
| **Do you use BMI and/or waist circumference and/or waist to height ratio within the criteria? (n=144)** |  |
| BMI | 122 (84.7) |
| Waist-to-Height ratio | 6 (4.2) |
| Waist circumference | 3 (2.1) |
| There is no limit or cut-off | 16 (11.1) |
| Individualised | 52 (36.1) |
| Other | 8 (5.6) |
| **What BMI range do you use? (n=122)** |  |
| BMI< 30kg/m2 | 25 (20.5) |
| BMI< 35kg/m2 | 66 (54.1) |
| BMI< 40kg/m2 | 31 (25.4) |
| Other | 21 (17.2) |
| **What waist-to-height ratio do you use? (n=6)** |  |
| ≥0.6 | 2 (33.3) |
| ≥0.7 | 2 (33.3) |
| ≥0.8 | 0 (0) |
| Other | 2 (33.3) |
| **What Waist circumference do you use? (n=3)** |  |
| < 94cm (men) | 0 (0) |
| < 108cm (men) | 2 (66.6) |
| < 80cm (women) | 0 (0) |
| < 88cm(women) | 2 (66.6) |
| Other | 1 (33.3) |
| **Does the referral criteria [for transplantation] differ within your transplanting centre e.g. between different surgeons? (n=212)** |  |
| Yes | 52 (24.5) |
| No | 69 (32.5) |
| Don’t know | 91 (42.9) |
| **From those that said referral criteria differed - What are the differences in referral criteria? (n=52)** |  |
| BMI | 37 (71.2) |
| Waist-to-height ratio | 7 (13.5) |
| Waist circumferences | 8 (15.4) |
| Individual surgical assessment | 37 (71.2) |
| Other | 1 (1.9) |
| **Do you have a separate transplant assessment pathway for people with obesity and diabetes (n=213)** |  |
| Yes | 8 (3.5) |
| No | 114 (53.5) |
| Don’t know | 91 (42.7) |
| **Do you know how many people, in percent, who are medically fit for kidney transplant listing not listed as a result of obesity/elevated BMI? (note: you can give an estimate if you do not know the exact number) (n=213)** |  |
| Yes | 38 (17.8) |
| No | 175 (82.2) |
|  |  |
| **Can you estimate how many in present (%) are not listed [for transplantation as a result of obesity/elevated BMI]? (n=38) median (IQR)** | 10.0 (7.3, 25.0) |
| **Do you have access to a service to help kidney donors to lose weight? (n=212)** |  |
| Yes | 52 (24.5) |
| No | 93 (43.9) |
| Don’t know | 67 (31.6) |

N, number; % percentage; BMI, body mass index; IQR, interquartile range; cm, centimetre; Kg/m^2^, kilograms per metre squared

**Table S9 Demographics about participants by devolved nations (n=227)**

| **Characteristics, n (%)** | **England** | | **Scotland** | | **Wales** | | **NI** | | **p-value** |
| --- | --- | --- | --- | --- | --- | --- | --- | --- | --- |
| **Gender, *n* (%)** |  |  |  |  |  |  |  |  | - |
| Male | 27 | (17.8) | 9 | (27.3) | 4 | (21.1) | 9 | (39.1) |  |
| Women | 124 | (81.6) | 24 | (72.7) | 14 | (73.7) | 14 | (60.9) |  |
| Prefer not to say | 1 | (0.7) | 0 | (0.0) | 1 | (5.3) | 0 | (0.0) |  |
| **Age, years (SD)** | 44.6 | 10.8 | 44.3 | 11.3 | 44.3 | 11.3 | 42.2 | 7.8 | - |
| **Ethnicity, *n* (%)** |  |  |  |  |  |  |  |  | - |
| Asian | 23 | (15.1) | 1 | (3.0) | 1 | (5.3) | 2 | (8.7) |  |
| Black | 2 | (1.3) | 0 | (0.0) | 0 | (0.0) | 0 | (0.0) |  |
| Mixed | 4 | (2.6) | 1 | (3.0) | 0 | (0.0) | 0 | (0.0) |  |
| Other | 1 | (0.7) | 0 | (0.0) | 0 | (0.0) | 0 | (0.0) |  |
| White | 118 | (77.6) | 31 | (93.9) | 17 | (89.5) | 21 | (91.3) |  |
| Prefer not to say | 4 | (2.6) | 0 | (0.0) | 1 | (5.3) | 0 | (0.0) |  |
| **Years since registration, mean (SD)** | 14.4 | 9.9 | 14.8 | 10.7 | 15.9 | 9.0 | 15.0 | 7.2 | - |
| **Role within the service, *n* (%)** |  |  |  |  |  |  |  |  | - |
| Dietitian | 57 | (37.5) | 10 | (30.3) | 5 | (26.3) | 5 | (21.7) |  |
| Nephrologist | 29 | (19.1) | 7 | (21.2) | 2 | (10.5) | 12 | (52.2) |  |
| Psychologist/Psychiatrist | 3 | (2.0) | 1 | (3.0) | 3 | (15.8) | 0 | (0.0) |  |
| Nurse | 35 | (23.0) | 3 | (9.1) | 5 | (26.3) | 6 | (26.1) |  |
| Surgeon | 6 | (3.9) | 6 | (18.2) | 2 | (10.5) | 0 | (0.0) |  |
| Physiotherapist | 6 | (3.9) | 0 | (0.0) | 2 | (10.5) | 0 | (0.0) |  |
| OT | 3 | (2.0) | 0 | (0.0) | 0 | (0.0) | 0 | (0.0) |  |
| Pharmacist | 5 | (3.3) | 1 | (3.0) | 0 | (0.0) | 0 | (0.0) |  |
| Counsellor | 2 | (1.3) | 0 | (0.0) | 0 | (0.0) | 0 | (0.0) |  |
| Other | 6 | (3.9) | 5 | (15.1) | 0 | (0.0) | 0 | (0.0) |  |
| **What patients do you support in your professional role, *n* (%)** |  |  |  |  |  |  |  |  | - |
| Haemodialysis | 143 | (94.1) | 26 | (78.8) | 17 | (89.5) | 22 | (95.7) |  |
| Peritoneal Dialysis | 91 | (59.9) | 16 | (48.5) | 13 | (68.4) | 18 | (78.3) |  |
| Transplant | 91 | (59.9) | 23 | (69.7) | 15 | (78.9) | 15 | (65.2) |  |
| Advanced Kidney Care | 103 | (67.8) | 21 | (63.6) | 9 | (47.4) | 17 | (73.9) |  |
| General Nephrology | 81 | (53.3) | 18 | (54.5) | 12 | (63.2) | 20 | (87.0) |  |
| Supportive care | 65 | (42.8) | 20 | (60.6) | 6 | (31.6) | 14 | (60.9) |  |
| Other | 10 | (6.6) | 3 | (9.1) | 3 | (15.8) | 0 | (0.0) |  |

**Table S10 Survey questions by devolved nations (n=227)**

| **Questions, *n* (%)** | **England** | | **Scotland** | | **Wales** | | **NI** | | **p-value** |
| --- | --- | --- | --- | --- | --- | --- | --- | --- | --- |
| **What are the challenges to helping these patients lose weight? *n* (%)** |  |  |  |  |  |  |  |  | Overall = 0.098 |
| Lack of time | 85 | 55.9 | 20 | 60.6 | 11 | 57.9 | 7 | 30.4 | 0.109 |
| Lack of financial investment/resources | 89 | 58.6 | 22 | 66.7 | 8 | 42.1 | 14 | 60.9 | 0.382 |
| Lack of expertise | 68 | 44.7 | 16 | 48.5 | 3 | 15.8 | 8 | 34.8 | 0.075 |
| Not seen as a priority in your department | 38 | 25.0 | 9 | 27.3 | 3 | 15.8 | 4 | 17.4 | 0.674 |
| Patients not interested | 77 | 50.7 | 16 | 48.5 | 15 | 78.9 | 9 | 39.1 | 0.063 |
| Lack of education or expertise within the area of obesity management | 83 | 54.6 | 19 | 57.6 | 6 | 31.6 | 10 | 43.5 | 0.198 |
| Lack of specialised services to refer to | 98 | 64.5 | 18 | 54.5 | 6 | 31.6 | 18 | 78.3 | 0.011 |
| Waiting list for obesity service referral | 91 | 59.9 | 25 | 75.8 | 12 | 63.2 | 8 | 34.8 | 0.022 |
| Other | 8 | 5.3 | 3 | 9.1 | 1 | 5.3 | 6 | 26.1 | 0.007 |
| **Do you have access to an obesity management service to help people with kidney failure lose weight? (CKD stage 5) *n* (%)** |  |  |  |  |  |  |  |  |  |
| Yes | 84 | 57.9 | 23 | 71.9 | 17 | 89.5 | 7 | 31.8 | 0.001 |
| No | 42 | 29.0 | 4 | 12.5 | 0 | 0.0 | 11 | 50.0 |  |
| Don’t know | 19 | 13.1 | 5 | 15.6 | 2 | 10.5 | 4 | 18.2 |  |
| **What services are available to patients to be referred to? (n=227) *n* (%)** |  |  |  |  |  |  |  |  |  |
| Individual weight management support | 58 | 38.2 | 17 | 51.5 | 13 | 68.4 | 4 | 17.4 | < 0.001 |
| Group weight management programme | 29 | 19.1 | 15 | 45.5 | 16 | 84.2 | 1 | 4.3 |  |
| Pharmacotherapy support | 30 | 19.7 | 3 | 9.1 | 2 | 10.5 | 1 | 4.3 |  |
| Bariatric surgery | 54 | 35.5 | 13 | 39.4 | 2 | 10.5 | 0 | 0.0 |  |
| Other | 7 | 4.6 | 3 | 9.1 | 0 | 0.0 | 3 | 13.0 |  |
| **Which healthcare professionals are involved with helping patients with obesity and kidney disease to lose weight (n=131) *n* (%)** |  |  |  |  |  |  |  |  |  |
| Specialist Dietitian e.g. renal or weight management | 133 | 87.5 | 32 | 97.0 | 19 | 100.0 | 21 | 91.3 | 0.003 |
| Specialist Psychologist e.g.,clinical psychologist, counsellor | 42 | 27.6 | 6 | 18.2 | 10 | 52.6 | 2 | 8.7 |  |
| Specialist Weight management consultant physician/Endocrinologist | 15 | 9.9 | 2 | 6.1 | 1 | 5.3 | 1 | 4.3 |  |
| Specialist Physical Activity Specialist e.g. Physiotherapy | 23 | 15.1 | 1 | 3.0 | 11 | 57.9 | 0 | 0.0 |  |
| Other | 20 | 13.2 | 2 | 6.1 | 0 | 0.0 | 1 | 4.3 |  |
| **How many patients have you referred to bariatric surgery in the last 12 months *n* (%)** |  |  |  |  |  |  |  |  |  |
| Yes | 64 | 44.8 | 13 | 40.6 | 2 | 10.5 | 5 | 22.7 | 0.012 |
| No | 79 | 55.2 | 19 | 59.4 | 17 | 89.5 | 17 | 77.3 |  |
| **Do you have any patients who have started GLP-1 receptor agonists? *n* (%)** |  |  |  |  |  |  |  |  |  |
| Yes | 57 | 39.9 | 7 | 21.9 | 5 | 26.3 | 6 | 27.3 | <0.001 |
| No | 20 | 14.0 | 16 | 50.0 | 4 | 21.1 | 11 | 50.0 |  |
| Don’t know | 66 | 46.2 | 9 | 28.1 | 10 | 52.6 | 5 | 22.7 |  |
| **Do you know the referral criteria at your centre or centre you refer to, if any, for listing patients for kidney transplantation in patients with obesity and kidney (n=214) *n* (%)** |  |  |  |  |  |  |  |  |  |
| Yes | 93 | 65.5 | 24 | 75.0 | 14 | 73.7 | 13 | 61.9 | 0.636 |
| No | 49 | 34.5 | 8 | 25.0 | 5 | 26.3 | 8 | 38.1 |  |
| **Do you use BMI and/or waist circumference and/or waist to height ratio within the criteria? *n* (%)** |  |  |  |  |  |  |  |  | Overall = 0.238 |
| BMI | 80 | 52.6 | 18 | 54.5 | 13 | 68.4 | 11 | 47.8 | 0.561 |
| Waist to Height ratio | 6 | 3.9 | 0 | 0.0 | 0 | 0.0 | 0 | 0.0 | 0.385 |
| Waist circumference | 3 | 2.0 | 0 | 0.0 | 0 | 0.0 | 0 | 0.0 | 0.682 |
| There is no limit or cut-off | 12 | 7.9 | 2 | 6.1 | 0 | 0.0 | 2 | 8.7 | 0.626 |
| Individualised | 32 | 21.1 | 11 | 33.3 | 4 | 21.1 | 5 | 21.7 | 0.497 |
| Other | 2 | 1.3 | 4 | 12.1 | 2 | 10.5 | 0 | 0.0 | 0.005 |
| **What BMI range do you use? *n* (%)** |  |  |  |  |  |  |  |  |  |
| BMI< 30kg/m2 | 15 | 9.9 | 5 | 15.2 | 5 | 26.3 | 0 | 0.0 | 0.156 |
| BMI< 35kg/m2 | 43 | 28.3 | 10 | 30.3 | 9 | 47.4 | 4 | 17.4 |  |
| BMI< 40kg/m2 | 18 | 11.8 | 7 | 21.2 | 2 | 10.5 | 4 | 17.4 |  |
| Other | 15 | 9.9 | 2 | 6.1 | 0 | 0.0 | 4 | 17.4 |  |
| **Does the referral criterion differ within your transplanting centre? *n* (%)** |  |  |  |  |  |  |  |  |  |
| Yes | 35 | 25.0 | 7 | 21.9 | 5 | 26.3 | 5 | 23.8 | 0.601 |
| No | 43 | 30.7 | 15 | 46.9 | 4 | 21.1 | 7 | 33.3 |  |
| Don’t know | 62 | 44.3 | 10 | 31.2 | 10 | 52.6 | 9 | 42.9 |  |

**Table S11 Demographics about participants by clinical role (n=227)**

| **Characteristics, n (%)** | **Medical** | | **Other clinical roles** | | **p-value** |  |
| --- | --- | --- | --- | --- | --- | --- |
| **Gender, *n* (%)** |  |  |  |  | - |  |
| Male | 37 | (32.2) | 12 | (10.7) |  |  |
| Women | 76 | (66.1) | 100 | (89.3) |  |  |
| Prefer not to say | 2 | (1.7) | 0 | (0.0) |  |  |
| **Age, years (SD)** | 47.6 | 9.4 | 41.4 | 10.6 |  |  |
| **Ethnicity, *n* (%)** |  |  |  |  | - |  |
| Asian | 19 | (17.4) | 8 | (6.2) |  |  |
| Black | 0 | (0.0) | 2 | (1.8) |  |  |
| Mixed | 2 | (1.7) | 3 | (2.7) |  |  |
| Other | 0 | (0.0) | 1 | (0.9) |  |  |
| White | 90 | (77.4) | 97 | (87.5) |  |  |
| Prefer not to say | 4 | (3.5) | 1 | (0.9) |  |  |
| **Years since registration, mean (SD)** | 18.4 | 9.4 | 10.8 | 8.3 | - |  |
| **What patients do you support in your professional role, *n* (%)** |  |  |  |  | - |  |
| Haemodialysis | 99 | (87.0) | 109 | (96.4) |  |  |
| Peritoneal Dialysis | 62 | (54.8) | 76 | (67.0) |  |  |
| Transplant | 74 | (64.3) | 70 | (62.5) |  |  |
| Advanced Kidney Care | 67 | (59.1) | 83 | (73.2) |  |  |
| General Nephrology | 61 | (53.9) | 70 | (61.6) |  |  |
| Supportive care | 48 | (42.6) | 57 | (50.0) |  |  |
| Other | 8 | (7.0) | 8 | (7.1) |  |  |

**Table S12 Survey questions by clinical roles (n=227)**

| **Questions, *n* (%)** | **Medical** | | **Other clinical roles** | | **p-value** |
| --- | --- | --- | --- | --- | --- |
| **What are the challenges to helping these patients lose weight? *n* (%)** |  |  |  |  | Overall = 0.237 |
| Lack of time | 54 | 47.0 | 69 | 61.6 | 0.051 |
| Lack of financial investment/resources | 63 | 54.8 | 70 | 62.5 | 0.362 |
| Lack of expertise | 55 | 47.8 | 40 | 35.7 | 0.064 |
| Not seen as a priority in your department | 23 | 20.0 | 31 | 27.7 | 0.174 |
| Patients not interested | 57 | 49.6 | 60 | 53.6 | 0.546 |
| Lack of education or expertise within the area of obesity management | 67 | 58.3 | 51 | 45.5 | 0.055 |
| Lack of specialised services to refer to | 79 | 68.7 | 61 | 54.5 | 0.013 |
| Waiting list for obesity service referral | 67 | 58.3 | 69 | 61.6 | 0.808 |
| Other | 8 | 7.0 | 10 | 8.9 | 0.582 |
| **Do you have access to an obesity management service to help people with kidney failure lose weight? (CKD stage 5) *n* (%)** |  |  |  |  |  |
| Yes | 65 | 58.6 | 66 | 61.7 | 0.157 |
| No | 26 | 23.4 | 31 | 29.0 |  |
| Don’t know | 20 | 18.0 | 10 | 9.3 |  |
| **What services are available to patients to be referred to? (n=227) *n* (%)** |  |  |  |  |  |
| Individual weight management support | 43 | 37.4 | 49 | 43.8 | 0.480 |
| Group weight management programme | 25 | 21.7 | 36 | 32.1 |  |
| Pharmacotherapy support | 17 | 14.8 | 19 | 17.0 |  |
| Bariatric surgery | 33 | 28.7 | 36 | 32.1 |  |
| Other | 9 | 7.8 | 4 | 3.6 |  |
| **Which healthcare professionals are involved with helping patients with obesity and kidney disease to lose weight (n=131) *n* (%)** |  |  |  |  |  |
| Specialist Dietitian e.g. renal or weight management | 105 | 91.3 | 100 | 89.3 | 0.187 |
| Specialist Psychologist e.g.,clinical psychologist, counsellor | 29 | 25.2 | 31 | 27.7 |  |
| Specialist Weight management consultant physician/Endocrinologist | 6 | 5.2 | 13 | 11.6 |  |
| Specialist Physical Activity Specialist e.g. Physiotherapy | 12 | 10.4 | 23 | 20.5 |  |
| Other | 9 | 7.8 | 14 | 12.5 |  |
| **How many patients have you referred to bariatric surgery in the last 12 months *n* (%)** |  |  |  |  |  |
| Yes | 47 | 42.7 | 37 | 34.9 | 0.238 |
| No | 63 | 57.3 | 69 | 65.1 |  |
| **Do you have any patients who have started GLP-1 receptor agonists? *n* (%)** |  |  |  |  |  |
| Yes | 41 | 37.3 | 34 | 32.1 | 0.678 |
| No | 26 | 23.6 | 25 | 23.6 |  |
| Don’t know | 43 | 39.1 | 47 | 44.3 |  |
| **Do you know the referral criteria at your centre or centre you refer to, if any, for listing patients for kidney transplantation in patients with obesity and kidney (n=214) *n* (%)** |  |  |  |  |  |
| Yes | 77 | 71.3 | 67 | 63.2 | 0.207 |
| No | 31 | 28.7 | 39 | 36.8 |  |
| **Do you use BMI and/or waist circumference and/or waist to height ratio within the criteria? *n* (%)** |  |  |  |  |  |
| BMI | 64 | (55.7) | 58 | 51.8 | 0.333 |
| Waist to Height ratio | 5 | (4.3) | 1 | 0.9 |  |
| Waist circumference | 1 | (0.9) | 2 | 1.8 |  |
| There is no limit or cut-off | 8 | (7.0) | 8 | 7.1 |  |
| Individualised | 30 | (26.1) | 22 | 19.6 |  |
| Other | 2 | (1.7) | 6 | 5.4 |  |
| **What BMI range do you use? *n* (%)** |  |  |  |  |  |
| BMI< 30kg/m2 | 9 | (7.8) | 16 | 14.3 | 0.187 |
| BMI< 35kg/m2 | 31 | (27.0) | 35 | 31.2 |  |
| BMI< 40kg/m2 | 20 | (17.4) | 11 | 9.8 |  |
| Other | 10 | (8.7) | 11 | 9.8 |  |
| **Does the referral criterion differ within your transplanting centre? *n* (%)** |  |  |  |  |  |
| Yes | 26 | (24.3) | 26 | 24.8 | <0.001 |
| No | 48 | (44.9) | 21 | 20.0 |  |
| Don’t know | 33 | (30.8) | 58 | 55.2 |  |

**Table S13 Demographics about participants comparing transplant vs referral centres (n=227)**

| **Characteristics, n (%)** | **Transplant Centre** | | **Referral Centre** | | **p-value** |
| --- | --- | --- | --- | --- | --- |
| **Gender, *n* (%)** |  |  |  |  | - |
| Male | 21 | (21.0) | 28 | (22.0) |  |
| Women | 79 | (79.0) | 97 | (76.4) |  |
| Prefer not to say | 0 | (0.0) | 2 | (1.6) |  |
| **Age, years (SD)** | 43.3 | 10.5 | 45.5 | 10.4 |  |
| **Ethnicity, *n* (%)** |  |  |  |  | - |
| Asian | 14 | (14.0) | 13 | (10.2) |  |
| Black | 1 | (1.0) | 1 | (0.8) |  |
| Mixed | 1 | (1.0) | 4 | (3.1) |  |
| Other | 1 | (1.0) | 1 | (0.8) |  |
| White | 80 | (80.0) | 107 | (84.3) |  |
| Prefer not to say | 3 | (3.0) | 2 | (1.6) |  |
| **Years since registration, mean (SD)** | 12.7 | 8.8 | 16.2 | 10.0 |  |
| **What patients do you support in your professional role, *n* (%)** |  |  |  |  | - |
| Haemodialysis | 92 | (92.0) | 116 | (91.3) |  |
| Peritoneal Dialysis | 66 | (66.0) | 72 | (56.7) |  |
| Transplant | 65 | (65.0) | 79 | (62.2) |  |
| Advanced Kidney Care | 74 | (74.0) | 76 | (59.8) |  |
| General Nephrology | 69 | (69.0) | 62 | (48.8) |  |
| Supportive care | 55 | (55.0) | 50 | (39.4) |  |
| Other | 9 | (9.0) | 7 | (5.5) |  |

**Table S14 Survey questions comparing transplant vs referral centres (n=227)**

| **Questions, *n* (%)** | **Transplant Centre** | | **Referral Centre** | | **p-value** |
| --- | --- | --- | --- | --- | --- |
| **What are the challenges to helping these patients lose weight? *n* (%)** |  |  |  |  |  |
| Lack of time | 49 | (49.0%) | 74 | (58.3%) | 0.892 |
| Lack of financial investment/resources | 60 | (60.0%) | 73 | (57.5%) |  |
| Lack of expertise | 38 | (38.0%) | 57 | (44.9%) |  |
| Not seen as a priority in your department | 23 | (23.0%) | 31 | (24.4%) |  |
| Patients not interested | 51 | (51.0%) | 66 | (52.0%) |  |
| Lack of education or expertise within the area of obesity management | 47 | (47.0%) | 71 | (55.9%) |  |
| Lack of specialised services to refer to | 65 | (65.0%) | 75 | (59.1%) |  |
| Waiting list for obesity service referral | 56 | (56.0%) | 80 | (63.0%) |  |
| Other | 10 | (10.0%) | 8 | (6.3%) |  |
| **Do you have access to an obesity management service to help people with kidney failure lose weight? (CKD stage 5) *n* (%)** |  |  |  |  |  |
| Yes | 56 | (57.7%) | 75 | (62.0%) | 0.714 |
| No | 28 | (28.9%) | 29 | (24.0%) |  |
| Don’t know | 13 | (13.4%) | 17 | (14.0%) |  |
| **What services are available to patients to be referred to? (n=227) *n* (%)** |  |  |  |  |  |
| Individual weight management support | 39 | (39.0%) | 53 | (41.7%) | 0.956 |
| Group weight management programme | 24 | (24.0%) | 37 | (29.1%) |  |
| Pharmacotherapy support | 17 | (17.0%) | 19 | (15.0%) |  |
| Bariatric surgery | 30 | (30.0%) | 39 | (30.7%) |  |
| Other | 6 | (6.0%) | 7 | (5.5%) |  |
| **Which healthcare professionals are involved with helping patients with obesity and kidney disease to lose weight (n=131) *n* (%)** |  |  |  |  |  |
| Specialist Dietitian e.g. renal or weight management | 90 | (90.0%) | 115 | (90.6%) | 0.678 |
| Specialist Psychologist e.g.,clinical psychologist, counsellor | 21 | (21.0%) | 39 | (30.7%) |  |
| Specialist Weight management consultant physician/Endocrinologist | 8 | (8.0%) | 11 | (8.7%) |  |
| Specialist Physical Activity Specialist e.g. Physiotherapy | 12 | (12.0%) | 23 | (18.1%) |  |
| Other | 10 | (10.0%) | 13 | (10.2%) |  |
| **How many patients have you referred to bariatric surgery in the last 12 months *n* (%)** |  |  |  |  |  |
| Yes | 40 | (41.2%) | 44 | (37.0%) | 0.523 |
| No | 57 | (58.8%) | 75 | (63.0%) |  |
| **Do you have any patients who have started GLP-1 receptor agonists? *n* (%)** |  |  |  |  |  |
| Yes | 34 | (35.1%) | 41 | (34.5%) | 0.993 |
| No | 23 | (23.7%) | 28 | (23.5%) |  |
| Don’t know | 40 | (41.2%) | 50 | (42.0%) |  |
| **Do you know the referral criteria at your centre or centre you refer to, if any, for listing patients for kidney transplantation in patients with obesity and kidney (n=214) *n* (%)** |  |  |  |  |  |
| Yes | 66 | (68.8%) | 78 | (66.1%) | 0.681 |
| No | 30 | (31.2%) | 40 | (33.9%) |  |
| **Do you use BMI and/or waist circumference and/or waist to height ratio within the criteria? *n* (%)** |  |  |  |  |  |
| BMI | 58 | (58.0%) | 64 | (50.4%) | 0.080 |
| Waist to Height ratio | 0 | (0.0%) | 6 | (4.7%) |  |
| Waist circumference | 1 | (1.0%) | 2 | (1.6%) |  |
| There is no limit or cut-off | 5 | (5.0%) | 11 | (8.7%) |  |
| Individualised | 20 | (20.0%) | 32 | (25.2%) |  |
| Other | 1 | (1.0%) | 7 | (5.5%) |  |
| **What BMI range do you use? *n* (%)** |  |  |  |  |  |
| BMI< 30kg/m2 | 12 | (12.0%) | 13 | (10.2%) | 0.480 |
| BMI< 35kg/m2 | 35 | (35.0%) | 31 | (24.4%) |  |
| BMI< 40kg/m2 | 15 | (15.0%) | 16 | (12.6%) |  |
| Other | 7 | (7.0%) | 14 | (11.0%) |  |
| **Does the referral criterion differ within your transplanting centre? *n* (%)** |  |  |  |  |  |
| Yes | 24 | (25.3%) | 28 | (23.9%) | 0.003 |
| No | 20 | (21.1%) | 49 | (41.9%) |  |
| Don’t know | 51 | (53.7%) | 40 | (34.2%) |  |

**Table S15 F-Scale for participant by devolved nations (n=210)**

| **Questionnaire** | **England** | | **Scotland** | | **Wales** | | **NI** | | **p-value** |
| --- | --- | --- | --- | --- | --- | --- | --- | --- | --- |
| Fat Phobia Scale (mean [SD]) | 3.4 | 0.5 | 3.4 | 0.3 | 3.3 | 0.4 | 3.7 | 0.6 | 0.002 |
| **Attitudes towards people living with obesity** n, (%) |  |  |  |  |  |  |  |  |  |
| Positive attitudes | 1 | (0.7) | 0 | (0.0) | 0 | (0.0) | 0 | (0.0) |  |
| Negative attitudes | 135 | (99.3) | 32 | (100.0) | 18 | (100.0) | 21 | (100.0) |  |
| **Categories of fat phobia** n, (%) |  |  |  |  |  |  |  |  |  |
| No Fat Phobia | 2 | (1.5) | 0 | (0.0) | 0 | (0.0) | 0 | (0.0) |  |
| Mild fat phobia | 88 | (64.7) | 20 | (62.5) | 14 | (77.8) | 9 | (42.9) |  |
| Moderate fat phobia | 38 | (27.9) | 12 | (37.5) | 4 | (22.2) | 11 | (52.4) |  |
| High fat phobia | 8 | (5.9) | 0 | (0.0) | 0 | (0.0) | 1 | (4.8) |  |

**Table S16 F-Scale for participant by clinical role (n=210)**

| **Questionnaire** | **Medical** | | **Other clinical roles** | | | **p-value** |
| --- | --- | --- | --- | --- | --- | --- |
| Fat Phobia Scale (mean [SD]) | 3.6 | 0.5 | 3.3 |  | 0.4 |  |
| **Attitudes towards people living with obesity** n, (%) |  |  |  |  |  |  |
| Positive attitudes | 0 | (0.0) | 1 |  | (1.0) |  |
| Negative attitudes | 103 | (100.0) | 103 |  | (99.0) |  |
| **Categories of fat phobia** n, (%) |  |  |  |  |  |  |
| No Fat Phobia | 1 | (1.0) | 1 |  | (1.0) |  |
| Mild fat phobia | 52 | (50.5) | 79 |  | (76.0) |  |
| Moderate fat phobia | 44 | (42.7) | 21 |  | (20.2) |  |
| High fat phobia | 6 | (5.8) | 3 |  | (2.9) |  |

**Table S17 F-Scale comparing transplant vs referral centres (n=210)**

| **Questionnaire** | **Transplant Centre** | | **Referral Centre** | | | **p-value** |
| --- | --- | --- | --- | --- | --- | --- |
| Fat Phobia Scale (mean [SD]) | 3.4 | 0.5 | 3.4 |  | 0.4 |  |
| **Attitudes towards people living with obesity** n, (%) |  |  |  |  |  |  |
| Positive attitudes | 0 | (0.0) | 1 |  | (0.9) |  |
| Negative attitudes | 93 | (100.0) | 113 |  | (99.1) |  |
| **Categories of fat phobia** n, (%) |  |  |  |  |  |  |
| No Fat Phobia | 0 | (0.0) | 2 |  | (1.8) |  |
| Mild fat phobia | 59 | (63.4) | 72 |  | (63.2) |  |
| Moderate fat phobia | 29 | (31.2) | 36 |  | (31.6) |  |
| High fat phobia | 5 | (5.4) | 4 |  | (3.5) |  |

**Table S18 Univariable regression outputs, exploring the predictors of the Fat phobia Scale**

| **Predictors** | Coefficient | 95% CI | p-value |
| --- | --- | --- | --- |
| **Age** | 0.00 | [-0.00, 0.01] | 0.494 |
| **Ethnicity** |  |  |  |
| White | (ref) |  | 0.004 |
| Other Ethnic Groups | 0.25 | [0.08, 0.41] |  |
| **Gender** |  |  |  |
| Male | (ref) |  | 0.108 |
| Female | -0.13 | [-0.28, 0.03] |  |
| **Role** |  |  |  |
| Nephrologist | (ref) |  | <0.001 |
| AHP | -0.23 | [-0.39, -0.08] |  |
| Nurse | 0.18 | [-0.01, 0.36] |  |
| Surgeon | -0.11 | [-0.37, 0.16] |  |
| Other | -0.17 | [-0.39,0.06] |  |
| **Transplant Centre** |  |  |  |
| Yes | (ref) |  | 0.540 |
| No | -0.04 | [-0.17, 0.09] |  |
| **Year of Experience** | 0.01 | [-0.00, 0.01] | 0.066 |

**Table S19 Multivariable regression outputs, exploring the predictors of the Fat phobia Scale**

| **Predictors** | Coefficient | 95% CI | p-value |
| --- | --- | --- | --- |
| **Ethnicity** |  |  |  |
| White | (ref) |  | 0.007 |
| Other Ethnic Groups | 0.22 | [0.06, 0.38] |  |
| **Role** |  |  |  |
| Nephrologist | (ref) |  | <0.001 |
| AHP | -0.22 | [-0.38, -0.05] |  |
| Nurse | 0.18 | [-0.00, 0.36] |  |
| Surgeon | -0.10 | [-0.36, 0.17] |  |
| Other | -0.16 | [-0.40,0.07] |  |
| **Year of Experience** | 0.00 | [-0.00, 0.01] | 0.001 |

Multivariable regression outputs, exploring predictors of fat phobia scale.

Predictors were entered into the multivariable regression if they were shown to have some association (p<0.1) in the univariable regression analyses.

1. Registry UR. *UK Renal Registry 27th Annual Report – data to 31/12/2023*. 2025. <https://ukkidney.org/audit-research/annual-report>

2. Stenvinkel P, Ikizler TA, Mallamaci F, Zoccali C. Obesity and nephrology: results of a knowledge and practice pattern survey. *Nephrol Dial Transplant*. Nov 2013;28 Suppl 4:iv99-104. doi:10.1093/ndt/gft193
